# Development of a new tissue injector for subretinal transplantation of human embryonic stem cell derived retinal pigmented epithelium

**DOI:** 10.1186/s40942-017-0095-6

**Published:** 2017-10-30

**Authors:** Rodrigo A. Brant Fernandes, Francisco R. Stefanini, Paulo Falabella, Michael J. Koss, Trent Wells, Bruno Diniz, Ramiro Ribeiro, Paulo Schor, Mauricio Maia, Fernando M. Penha, David R. Hinton, Yu-Chong Tai, Mark Humayun

**Affiliations:** 1 Department of Ophthalmology, USC Roski Eye Institute, Keck School of Medicine, University of Southern California, Los Angeles, CA USA; 2 Department of Ophthalmology and Visual Sciences, Federal University of São Paulo, Rua Botucatu, 822, São Paulo, SP 04023-062 Brazil; 3 Augenzentrum Nymphenburger Hoefe, Herzog Carl Theodor Augenklinik, Munich, Germany; 4 Fundação Universidade Regional de Blumenau, Blumenau, Santa Catarina Brazil; 5 Electrical Engineering, California Institute of Technology, Pasadena, CA USA; 6 Department of Pathology, Keck School of Medicine, University of Southern California, Los Angeles, CA USA; 7 USC Institute for Biomedical Therapeutics, Keck School of Medicine, University of Southern California, Los Angeles, CA USA

**Keywords:** Transplantation, Stem cells, Parylene, Retinal pigment epithelium, Tissue injector, Macular degeneration

## Abstract

**Background:**

Subretinal cell transplantation is a challenging surgical maneuver. This paper describes the preliminary findings of a new tissue injector for subretinal implantation of an ultrathin non-absorbable substrate seeded with human embryonic stem cell-derived retinal pigment epithelium (hESC-RPE).

**Methods:**

Ultrathin Parylene-C substrates measuring 3.5 mm × 6.0 mm seeded with hESC-RPE (implant referred to as CPCB-RPE1) were implanted into the subretinal space of 12 Yucatan minipigs. Animals were euthanized immediately after the procedure and underwent spectral domain optical coherence tomography (SD-OCT) and histological analysis to assess the subretinal placement of the implant. Evaluation of the hESC-RPE cells seeded on the substrate was carried out before and after implantation using standard cell counting techniques.

**Results:**

The tissue injector delivered the CPCB-RPE1 implant through a 1.5 mm sclerotomy and a 1.0–1.5 mm retinectomy. SD-OCT scans and histological examination revealed that substrates were precisely placed in the subretinal space, and that the hESC-RPE cell monolayer continued to cover the surface of the substrate after the surgical procedure.

**Conclusion:**

This innovative tissue injector was able to efficiently deliver the implant in the subretinal space of Yucatan minipigs, preventing significant hESC-RPE cell loss, minimizing tissue trauma, surgical complications and postoperative inflammation.

## Background

Age-related macular degeneration (AMD) is the leading cause of severe visual loss and legal blindness in the elderly population [1, 2]. Although anti-angiogenic therapies have been developed to treat exudative AMD [3], there is no effective treatment for dry AMD, specially at its end stage, namely, geographic atrophy [4, 5]. Previous studies have demonstrated that dysfunction and/or death of retinal pigment epithelium (RPE) cells play a critical role in the pathophysiology of dry AMD and that RPE transplantation has the potential to halt further degeneration and restore visual function [6, 7].

Stem cells have the capacity to differentiate and replace damaged cells, providing an unlimited source of RPE cells for transplantation purposes [8]. In normal retinas, RPE cells consist of a polarized monolayer and embryonic stem cell-derived retinal pigment epithelium (hESC-RPE) cells cultured on an ultrathin substrate (e.g. Parylene-C) have similar characteristics. Although ultrathin substrates are theoretically good scaffolds for subretinal transplantation due to their permeability, they are usually soft and malleable. Their implantation often requires challenging surgical maneuvers, resulting in a large retinectomy associated with damage to the surrounding tissue.

The present study demonstrates a new tissue injector designed to perform subretinal transplantation of Parylene-C substrates seeded with hESC-RPE cells (implant referred to as CPCB-RPE1) in a safe and reproducible procedure, leading to minimal damage to the host retina as well as preventing significant hESC-RPE cell loss during the implantation.

## Methods

### Mesh membrane

**Fig. 1:**
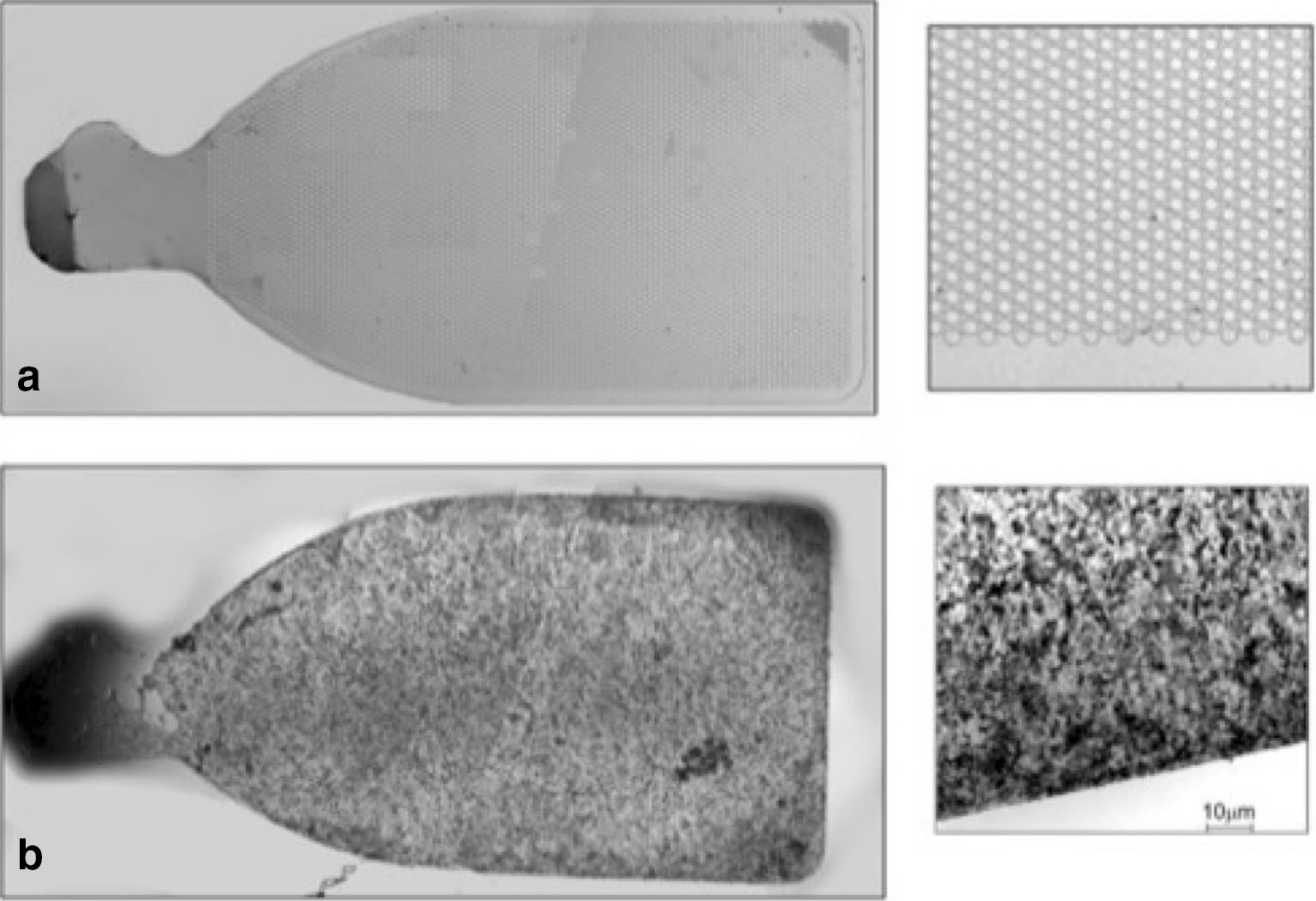
A 3.5 × 6 mm implant with 2 mm handle. **a** Unseeded. **b** Seeded with hESC-RPE

A mesh-supported sub-micron Parylene-C membrane (MSPM) is a class VI material (i.e. implant grade) comprised of a 0.30 μm thick Parylene-C membrane and a 6.0 μm thick supporting mesh (Fig. 1). The MSPM surface was seeded with hESC-RPE cells and treated with oxygen plasma. Parylene-C was provided by Specialty Coating Systems (Indianapolis, IN, USA), while the MSPM was provided by the California Institute of Technology (Los Angeles, CA, USA). Sub-micron Parylene-C is ultrathin, and its molecular weight, exclusion limit, and permeability are similar to the Bruch’s membrane [9–11] and, therefore, it is considered a good candidate for its replacement. Additionally, Parylene-C demonstrates low potential harmful effects in the subretinal environment [12].

This configuration allows for the formation of a polarized, confluent, functional monolayer of RPE cells, provides the mechanical support required for surgical implantation, and promotes the reciprocal exchange of nutrients and waste products between the RPE and choroid.

### Cell culture for implants

Human embryonic stem cells (Wicell, Madison, WI, USA) were spontaneously differentiated into RPE cells as described previously [11]. These hESC-RPE cells were cultured and maintained in serum-free medium X-VIVO 10 Lonza (Walkersville, Maryland, MD, USA) on Synthemax plates (Corning, New York, NY, USA). Based on staining with RPE markers, cultures showed over 95% of purity [13].

In the second passage, hESC-RPE cells were dissociated with trypsin (TrypLE—Invitrogen, Carlsbad, CA, USA) and seeded on MSPM films (3.5 mm × 6 mm) coated with vitronectin (BD Biosciences Franklin Lakes, NJ, USA) at a cell density of 10^5^/cm^2^. Cells were maintained in culture on the ultrathin parylene substrates for 4 weeks with the medium changed twice weekly.

A post mortem study showed a mean density of approximately 5000 RPE cells/mm^2^ on the macular area [14]. Based on the dimensions of the scaffolds, and the mean density of cells on the substrate (6200 RPE cells/mm^2^) individual CPCB-RPE1 implants had approximately a total of 125,000 cells. (Fig.  1).

### Tissue injector

**Fig. 2:**
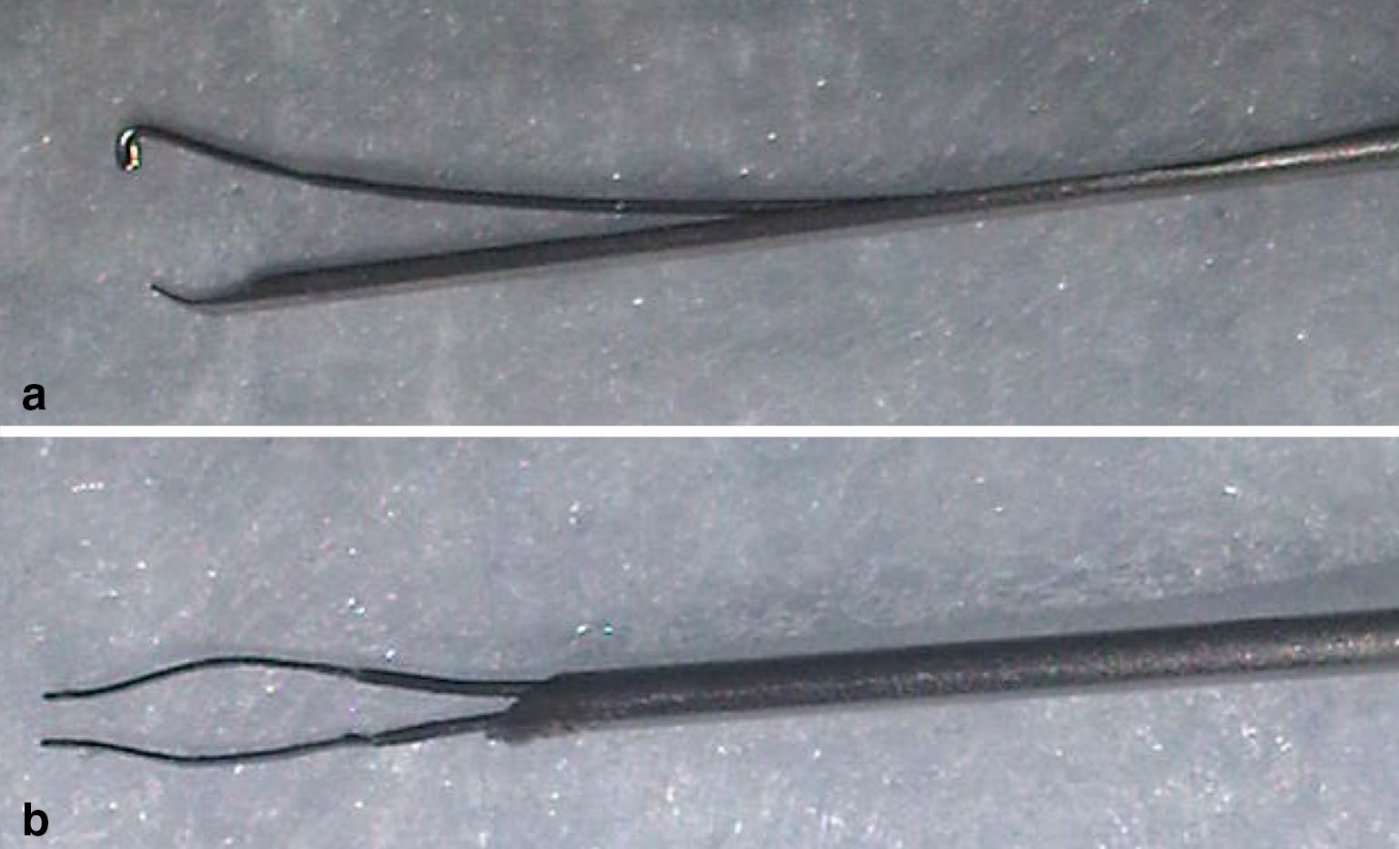
Comparison between the 15 gauge **a** versus 17 gauge **b** tissue injector. Tip design improvement prevent implant damage

**Fig. 3:**
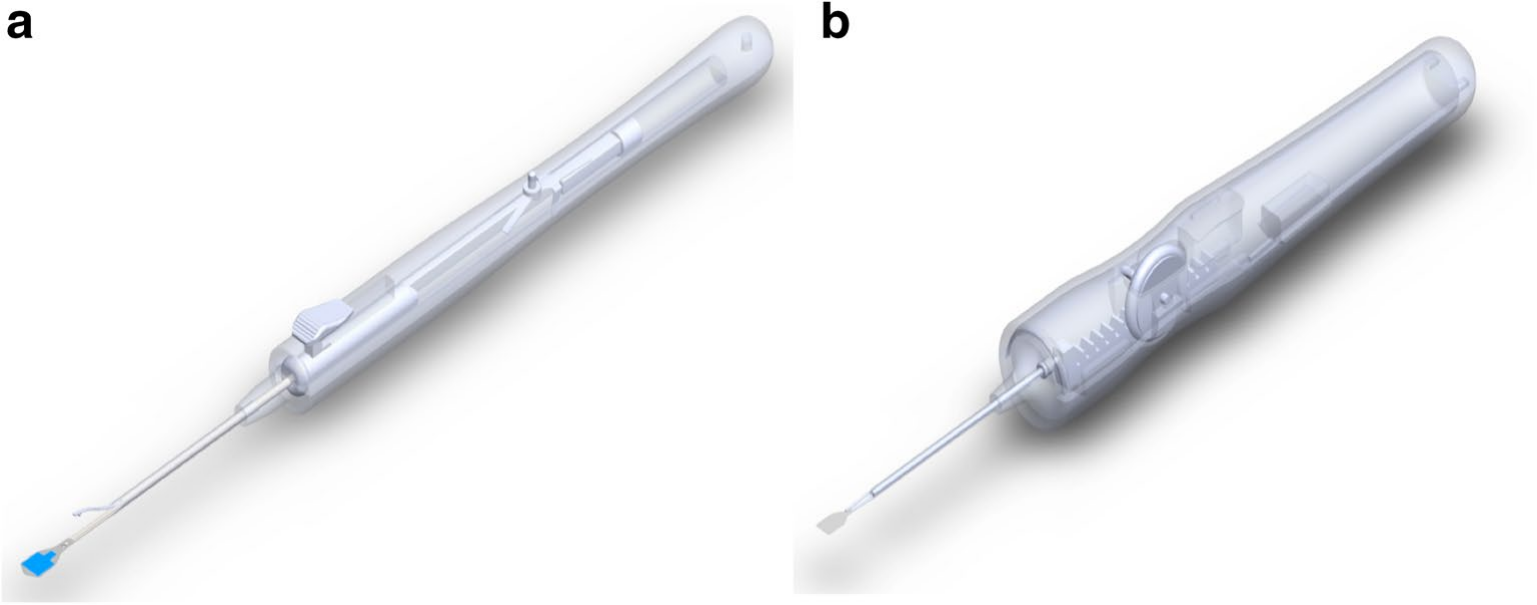
Injector design: previous version (**a**) versus latest version (**b**)

A first set of surgeries was performed in Yucatan minipigs using a 15-gauge prototype tissue injector to deliver hESC-RPE monolayer seeded over 4.5 mm × 4.5 mm Parylene-C substrates into their subretinal space (unpublished data). Based on this preliminary experience, the size of the substrate was reduced to 3.5 mm × 6 mm in order to decrease the diameter of the surgical injector and, consequently, the required sclerotomy and retinectomy (Fig. 2). The reduction in the size of the implant is consistent with the size of the macular area in order to maintain the same functionality. A new tissue injector prototype of 17-gauge was developed to handle this smaller substrate (Fig. 3).

**Fig. 4:**
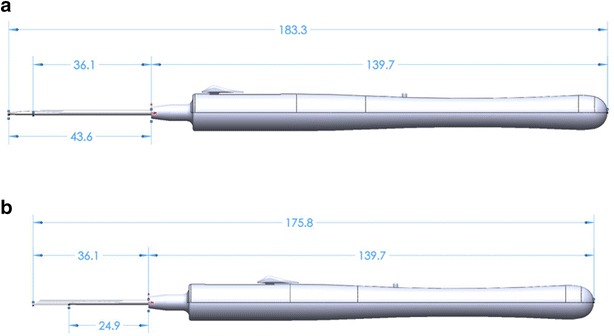
Tissue injector, version one shown in the extended and retracted position. The diameter of the tool measures 11.4 mm at the largest cross section and has an overall length of 175.8 mm when retracted (**b**) and 183.3 mm when fully extended (**a**). The tube measures 1.59 mm ID × 1.83 mm OD and has a length of 36.1 mm as measured from the tip of the tool’s body

**Fig. 5:**
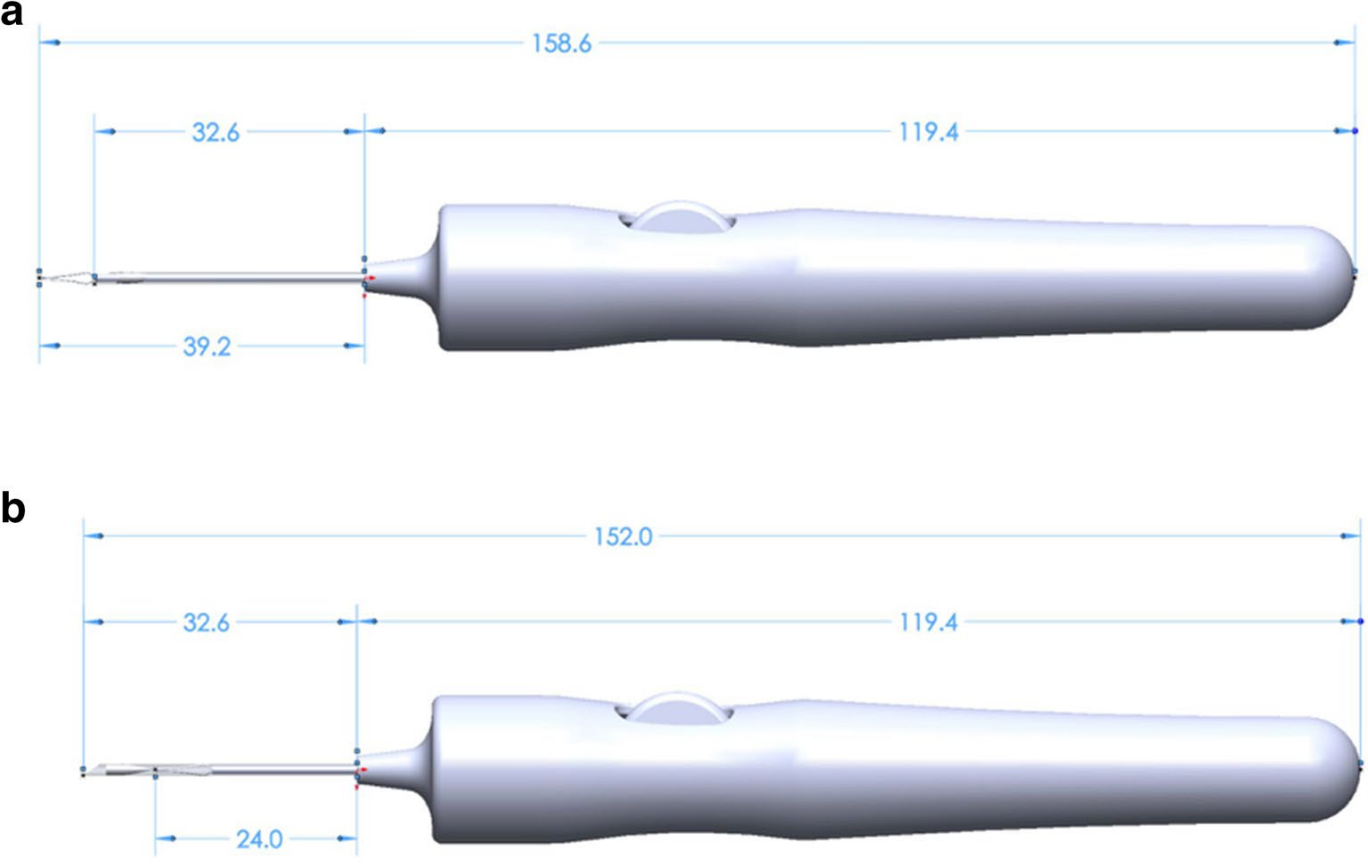
Tissue injector, version two shown in the extended and retracted position. The diameter of the tool measures 17.8 mm at the largest cross section and has an overall length of 152.0 mm when retracted (**b**) and 158.6 mm when fully extended (**a**). The tube measures 1.27 mm ID × 1.47 mm OD and has a length of 32.6 mm as measured from the tip of the tool’s body

The two versions of the tissue injector tools are shown in Figs. 4 and 5 with dimensions referenced for the major components of the injector. Dimensions are given for the extended and retracted positions to store and protect the membrane during insertion and deliver the substrate to the eye respectively.

Version 1 of the tissue insertion consisted of an overall length of 175.8 mm when retracted and 183.3 mm when extended. The tool length was driven by the primary need to store the forceps and substrate completely during retraction and a secondary need to remain short enough that it would not impede ergonomics of the surgeon during use. During the iteration between the two versions, both the overall membrane length and forceps length were shortened, allowing the length of the tube at the front and the tool’s body to be shortened by 3.5 mm. The new mechanism and the reduced length of the tube then allowed the body to also be shortened an additional 20.3 mm. Leading to an overall length reduction of 23.8 mm between versions with a retracted length of 152.0 mm and extended length of 158.6 mm.

The diameter of the tools at the largest cross section was increased between version iterations from 11.4 mm at the largest cross section in version one, up to a diameter of 17.8 mm in version two. The increased diameter between versions of the tool was driven by both the larger size of the internal mechanism and user feedback during experiments that a larger diameter would be more ergonomic for handling during procedures.

The internal lumen circumference must correspond to the width of the MSPM in order to fold the substrate without overlapping its edges inside of the instrument. Any overlap of the substrate edges could lead to loss of cells from the substrate.

The CPCB-RPE1 implant consisted of a rectangular portion with rounded edges intended to support the seeded hESC-RPE cells, and a 2 mm handle to allow its manipulation with a standard intraocular forceps and the tissue injector. A round marker on the side of the handle was added to identify which side was seeded with cells (marker always facing right during seeding and implantation).

### Surgical technique

In order to verify the safety and reproducibility of the surgical procedure for future application in human eyes, we have assessed the surgical tools, as well as the surgical techniques to deliver the implant into the subretinal space of 12 Yucatan minipigs. This animal model was chosen because the size and shape of the eye are very similar to human eyes, and previous studies showed that subretinal implantation in this model was a feasible procedure [12].

All experiments were performed in compliance with the ARVO statement of the use of Animals in Ophthalmic and Vision Research under a protocol approved by the Animal Care and Use Committee (IACUC) at the University of Southern California.

**Fig. 6:**
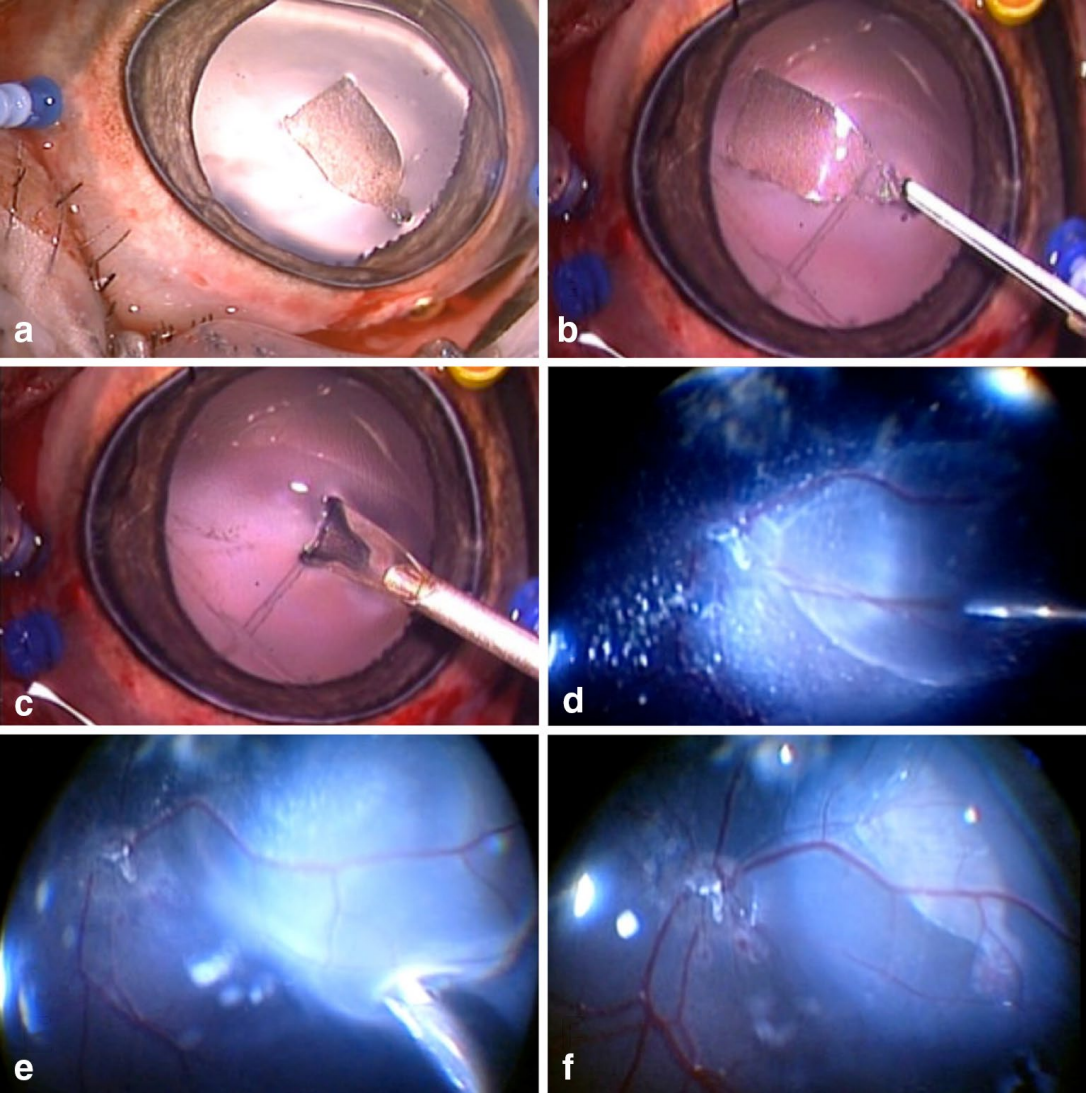
**a** Anterior segment and lens status during and after the surgical procedure. **b** Injector forceps holding the substrate by the handle. **c** Injector folding the substrate. **d** Injection of saline in the subretinal space. **e** Subretinal unfolding and placement. **f** Posterior pole aspect at the end of the surgical procedure

The surgical technique of implantation of the MPSM seeded with hESC-RPE consisted of Pars Plana Vitrectomy (PPV), a 1 mm retinectomy anterior to the equator followed by the placement of the substrate seeded with cells under the retina using the novel tissue injector (Fig. 6).

Pigs were submitted to general anesthesia using Xylazine/Telazol 1.1–2.2 mg/kg; Glycopyrrolate 0.01–0.04 mg/kg; SEVOfluarane/Isoflurane 1.0–3.5%; Buprenorphine 0.05–0.1 mg/kg; Rimadyl 2–4 mg/kg, conducted by a trained veterinary staff under veterinary supervision. The left eye was dilated using 1% tropicamide and phenylephrine eye drops. Proper asepsis with povidone iodine 10% was applied to the area surrounding the eye and the entire surgical field using 1% betadine drops instilled on the eye.

The eye was draped in a sterile surgical field and an eyelid speculum was applied. Lateral canthotomies and proper hemostasis were performed temporally to facilitate surgical manipulation. Three 25-gauge trocars were placed at 3.5 mm from the limbus as well as two 29-gauge chandeliers (Synergetics TM, USA), connected to the Photon 2 illumination system were used (Synergetics TM, USA).

Pars plana vitrectomy (PPV) was performed by a core vitrectomy using 600 mmHg vacuum, 5000 cuts/min, using the Stellaris PC Surgical system (Bausch and Lomb, USA), and a 25 gauge vitrectomy probe (Bausch and Lomb, USA). The Zeiss Opmi Microscope (Zeiss, Germany) was used with the Oculus Binocular Indirect Ophthalmic Microscope wide-angle visual system (BIOM System, USA). Staining of the posterior hyaloid was performed using 0.3 ml of triamcinolone acetonide (Triesence^®^ 40 mg/ml, Alcon, Inc., Fort Worth, TX, USA) followed by posterior hyaloid detachment using an aspiration rate of 200 mmHg until no residual vitreous (shown by triamcinolone) was observed at the retinal surface.

A complete vitrectomy was performed including 360° vitreous base shaving, followed by inspection of the vitreous base in order to rule out retinal tears.

After vitreous shaving, the posterior pole and superior retina was detached using a 41-gauge needle injection (Bausch and Lomb, USA) of approximately 1 ml of balanced saline solution (BSS) starting near the optic nerve head, creating a retinal bleb. Endodiathermy was performed anterior to the equator outside of the vascular arcades, comprising 1 mm, in a linear fashion, followed by retinectomy performed with the vitrectomy probe. A superior nasal sclerotomy was enlarged to 1.5 mm, allowing the tissue injector to place the seeded substrate under the retina, close to the optic nerve (Fig. 6).

Perfluorocarbon liquid (PFO) was slowly injected on the retinal surface to reattach the retina—filling the entire posterior pole—until subretinal fluid was completely displaced from the subretinal space. The 1.5 mm temporal sclerotomy was sutured using Vicryl 6.0. Endophotocoagulation was performed on the edges of the retinectomy, with at least three rows, followed by fluid-air exchange, and then the eye was filled with silicone oil 1000 centistokes Oxane™ (Bausch and Lomb, USA). Finally, the eye was patched with ointment containing corticosteroids and antibiotics.

The lens and anterior segment remained untouched during the procedure in order to prevent media opacities.

All study animals were euthanized immediately after the surgical procedure and imaging with 0.5 ml of pentobarbitol sodium 390 mg and phenytoin sodium 50 mg (Euthasol, Virbac AH, Inc, Forth Worth, TX, USA).

### Post-operative analysis

The implanted animals were evaluated before and immediately after the surgical procedure with the Heidelberg Spectralis™ SD-OCT (Heidelberg Engineering, Germany).

The imaging protocol was comprised of volume scans performed with SD-OCT, fluorescein angiogram (FA), fundus autofluorescence (FAF), color fundus photographs and Infrared (IR) images.

Color fundus photographs were taken of both eyes. IR images, FAF and FA were taken within 3 min of acquiring time (Heidelberg Spectralis, HRA-OCT) in both eyes. SD-OCT images (Heidelberg Spectralis-HRA) were also taken for both eyes using the following protocol: (1) High definition scans through the optic nerve; (2) volume scans (30 × 30) centered on the optic nerve; (3) volume scans (30 × 30) of the temporal retina with the optic nerve on the edge of the scan; (4) nerve fiber layer scans centered on the optic nerve.

### Histological evaluation and immunohistochemistry

After implantation, animals were euthanized and the enucleated eyes were fixed in Davidson’s solution for 24 h. For light microscopy, the tissue was stained with Hematoxylin and Eosin (HE) and evaluated using the M500 microscope (Leica, Wetzlar, Germany). Immunohistochemistry analysis was performed with anti-TRA-1-85, a human-specific cell marker used to identify the transplanted cells, and DAPI (4′,6-diamidino-2-phenylindole) staining, which labels both transplanted and host nuclei.

## Results

### Surgical results

Twelve Yucatan minipigs underwent surgical implantation of the CPCB-RPE1 implant in the subretinal space. The surgical technique was shown to be safe and reproducible, with no severe intraoperative complications, such as unplanned retinal detachment or iatrogenic retinal tears, significant retinal or choroidal bleeding, nor misdelivery of the implant. The lens was spared in all surgeries, without inadvertent touch and no opacity were noted during or immediately after the procedure.

**Fig. 7:**
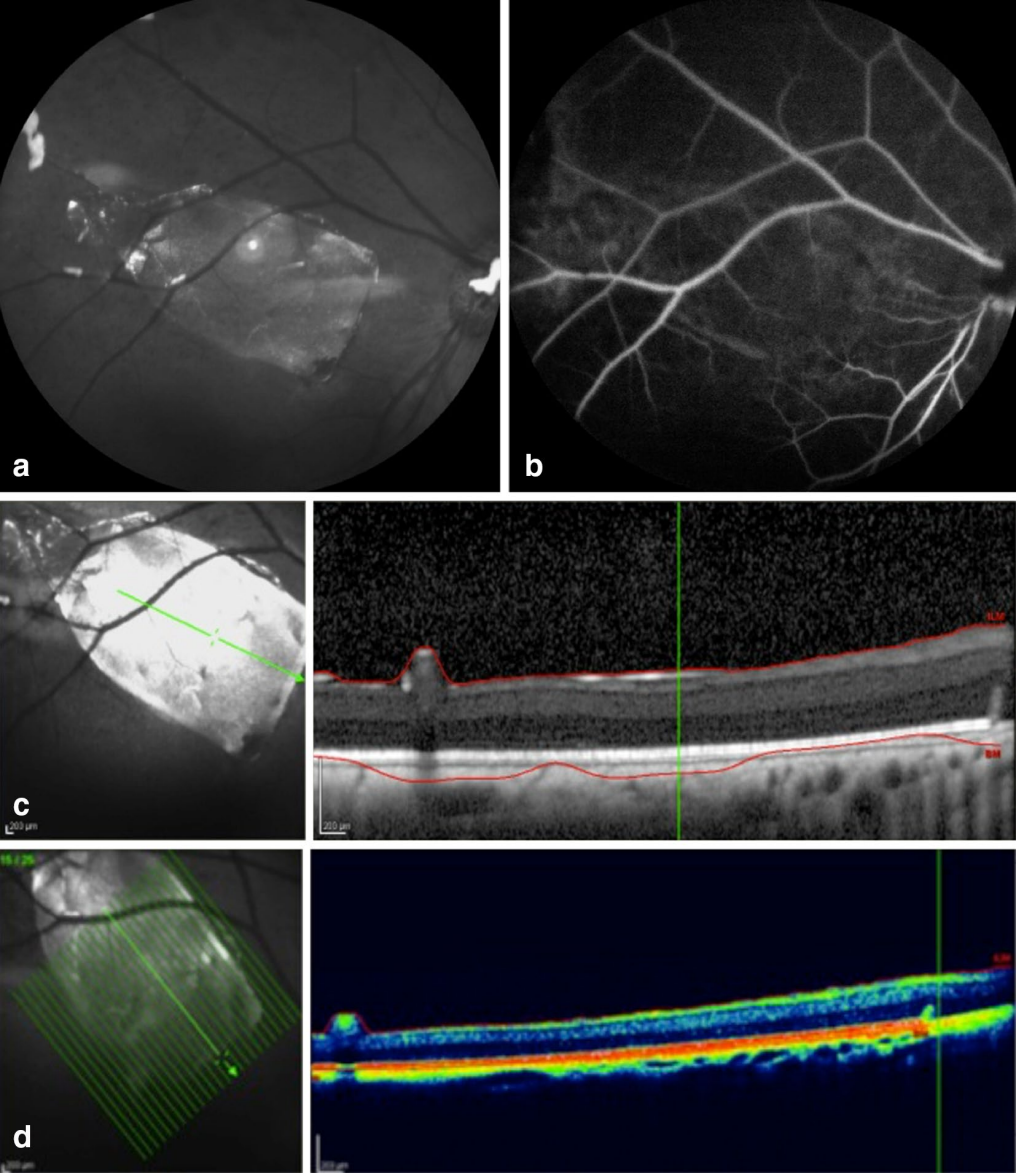
**a** Infrared image showing subretinal implant location. **b** Fluorescein angiography showing blockage of the fluorescein where the implant was placed. SD-OCT observed in **c** black and white; **d** colored, the subretinal location of the implant

Imaging of the posterior pole was performed in the immediate postoperative period, and, in all cases, the implant was clearly visualized in color fundus photographs as well as IR images, showing successful delivery. In FA, the implant was easily identified by fluorescence blockage of the choroid circulation, and SD-OCT B-scans showed adequate flat positioning of the implants in the subretinal space in all subjects, with no apparent damage to the retina (Fig. 7).

### Histological analysis

**Fig. 8:**
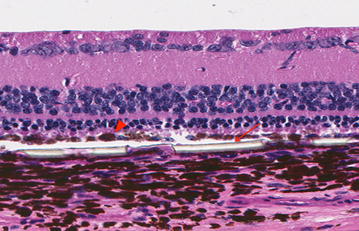
HE stained section through an implant removed shortly after implantation. Substrate placed in the subretinal space, with minimal damage to the retinal layers, photoreceptors intact. Implant thick regions shown with arrow; hESC-RPE on surface of implant shown with arrowhead

HE microscopic analysis demonstrated that all implants were unbroken and correctly placed underneath the retina, associated with only minor damage to the associated retinal tissues (Fig. 8).

**Fig. 9:**
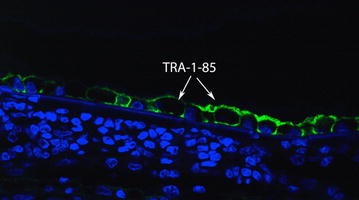
Immunohistochemistry analysis: anti-TRA-1-85, a human-specific cell marker, was used to identify the transplanted hESC-RPE (arrows)

The hESC-RPE cells that covered the surface of the implant were present in a monolayer in all operated eyes, as shown by immunohistochemistry (Fig. 9). No TRA-1-85 + human cells were found underneath the implant or within the retina, suggesting that there was no significant cell dispersion or migration during the delivery.

## Discussion

The rationale behind subretinal transplantation of a monolayer of hESC-RPE cells cultured over a scaffold prompts the need for the development of a unique surgical technique. Developing a new tissue injector tool to improve usability, reproducibility, performance and ultimately surgical safety was essential for the advancement of this investigational therapy. Incisions needed to be reduced and, therefore, substrate dimensions had to be resized in order to fit perfectly inside the cannula without overlapping its edges.

These requirements resulted in the design of a smaller tissue injector tool, resembling a standard retinal forceps. It allowed for the subretinal unfolding and positioning of the substrate with a single instrument, making the procedure easier, more reliable and reproducible. When compared to procedures performed previously with a larger tissue injector prototype (unpublished data), surgical time decreased from approximately 90–50 min on average, and minor procedure-related complications (e.g. small retinal bleeding during retinectomy) seemed to be less frequent.

This set of surgeries demonstrated a successful subretinal delivery of the implant without major complications. Additionally, the lens and the anterior segment were spared from trauma, decreasing the associated inflammation secondary to the lensectomy, which allowed for a clear view of the posterior pole during the procedure and in the immediate postoperative period. The surgical technique showed reproducibility and lack of complications, essential requirements for moving forward to clinical trials.

This study is limited by the small number of animals, and by the lack of long term follow up to access cell survival and functionality of the implant (e.g. phagocytosis of the photoreceptor outer segments by the hESC-RPE) [15, 16]. Regarding the newly designed tissue injector, although a smaller gauge was achieved, a 17-gauge instrument is still significantly large for eye surgeries. Decreasing the dimensions of the injector, and consequently the size of the sclerotomies and retinectomy, might lead to an even more controlled procedure. A new generation of this tool is under development.

The 17-gauge injector device, however, was shown to be useful, easy to handle and safe. It has been designed to deliver the CPCB-RPE1 implant in the subretinal space, however, this tool may be helpful in other surgical techniques with different kinds of substrates.

## Conclusion

In conclusion, the surgical procedure using this innovative device was reproducible and safe in a large animal model, causing minimal damage to the transplanted cells as well as to the host tissue. Additional studies in animal and human eyes are still necessary to further validate these findings.

## Abbreviations

hESC-RPE: human embryonic stem cell-derived retinal pigment epithelium
CPCB-RPE1: ultrathin Parylene-C membrane seeded with hESC-RPE
SD-OCT: spectral domain optical coherence tomography
RPE: retinal pigment epithelium
AMD: age-related macular degeneration
FAF: fundus autofluorescence
FA: fluorescein angiogram
IR: infrared (images)

## References

[1] Sarks SH (1976). Ageing and degeneration in the macular region: a clinico-pathological study. Br J Ophthalmol.

[2] Gehrs KM, Anderson DH, Johnson LV, Hageman GS (2006). Age-related macular degeneration—emerging pathogenetic and therapeutic concepts. Ann Med.

[3] Michels S, Rosenfeld PJ, Puliafito CA, Marcus EN, Venkatraman AS (2005). Systemic bevacizumab (Avastin) therapy for neovascular age-related macular degeneration twelve-week results of an uncontrolled open-label clinical study. Ophthalmology.

[4] Ambati J, Ambati BK, Yoo SH, Ianchulev S, Adamis AP (2003). Age-related macular degeneration: etiology, pathogenesis, and therapeutic strategies. Surv Ophthalmol.

[5] Evans J (2008). Antioxidant supplements to prevent or slow down the progression of AMD: a systematic review and meta-analysis. Eye (Lond).

[6] Glenn JV, Mahaffy H, Wu K (2009). Advanced glycation end product (AGE) accumulation on Bruch’s membrane: links to age-related RPE dysfunction. Invest Ophthalmol Vis Sci.

[7] Dorey CK, Wu G, Ebenstein D, Garsd A, Weiter JJ (1989). Cell loss in the aging retina. Relationship to lipofuscin accumulation and macular degeneration. Invest Ophthalmol Vis Sci.

[8] da Cruz L, Chen FK, Ahmado A, Greenwood J, Coffey P (2007). RPE transplantation and its role in retinal disease. Prog Retin Eye Res.

[9] Lu B, Zhu D, Hinton D, Humayun MS, Tai YC (2012). Mesh-supported submicron parylene-C membranes for culturing retinal pigment epithelial cells. Biomed Microdevices.

[10] Lee CJ, Vroom JA, Fishman HA, Bent SF (2006). Determination of human lens capsule permeability and its feasibility as a replacement for Bruch’s membrane. Biomaterials.

[11] Zhu D, Deng X, Spee C (2011). Polarized secretion of PEDF from human embryonic stem cell-derived RPE promotes retinal progenitor cell survival. Invest Ophthalmol Vis Sci.

[12] Montezuma SR, Loewenstein J, Scholz C, Rizzo JF (2006). Biocompatibility of materials implanted into the subretinal space of Yucatan pigs. Invest Ophthalmol Vis Sci.

[13] Koss MJ, Falabella P, Stefanini FR (2016). Subretinal implantation of a monolayer of human embryonic stem cell-derived retinal pigment epithelium: a feasibility and safety study in Yucatan minipigs. Graefe’s Arch Clin Exp Ophthalmol.

[14] Del Priore LV, Tezel TH, Kaplan HJ (2006). Maculoplasty for age-related macular degeneration: reengineering Bruch’s membrane and the human macula. Prog Retin Eye Res..

[15] Hu Y, Liu L, Lu B (2012). A novel approach for subretinal implantation of ultrathin substrates containing stem cell-derived retinal pigment epithelium monolayer. Ophthalmic Res.

[16] Diniz B, Thomas P, Thomas B (2013). Subretinal implantation of retinal pigment epithelial cells derived from human embryonic stem cells: improved survival when implanted as a monolayer. Invest Ophthalmol Vis Sci.

